# 10.321/eid0805.Typical and Atypical Enteropathogenic *Escherichia coli*

**DOI:** 10.3201/eid0805.010385

**Published:** 2002-05

**Authors:** Luiz R. Trabulsi, Rogéria Keller, Tânia A. Tardelli Gomes

**Affiliations:** * Laboratório Especial de Microbiologia, Instituto Butantan, São Paulo, Brazil; † Universidade Federal de São Paulo, São Paulo, Brazil

## Abstract

Typical and atypical enteropathogenic *Escherichia coli* (EPEC) strains differ in several characteristics. Typical EPEC, a leading cause of infantile diarrhea in developing countries, is rare in industrialized countries, where atypical EPEC seems to be a more important cause of diarrhea. For typical EPEC, the only reservoir is humans; for atypical EPEC, both animals and humans can be reservoirs. Typical and atypical EPEC also differ in genetic characteristics, serotypes, and virulence properties. Atypical EPEC is more closely related to Shiga toxin–producing *E. coli* (STEC), and like STEC these strains appear to be emerging pathogens.

Enteropathogenic *Escherichia coli* (EPEC) is a leading cause of infantile diarrhea in developing countries. In industrialized countries, the frequency of these organisms has decreased, but they continue to be an important cause of diarrhea (*1*). The central mechanism of EPEC pathogenesis is a lesion called attaching and effacing (A/E), which is characterized by microvilli destruction, intimate adherence of bacteria to the intestinal epithelium, pedestal formation, and aggregation of polarized actin and other elements of the cytoskeleton at sites of bacterial attachment (Figure 1). The fluorescent actin staining test allows the identification of strains that produce A/E lesions, through detection of aggregated actin filaments beneath the attached bacteria (*3*). Ability to produce A/E lesions has also been detected in strains of Shiga toxin–producing *E. coli* (enterohemorrhagic *E. coli* [EHEC]) and in strains of other bacterial species (1).

**Figure 1:**
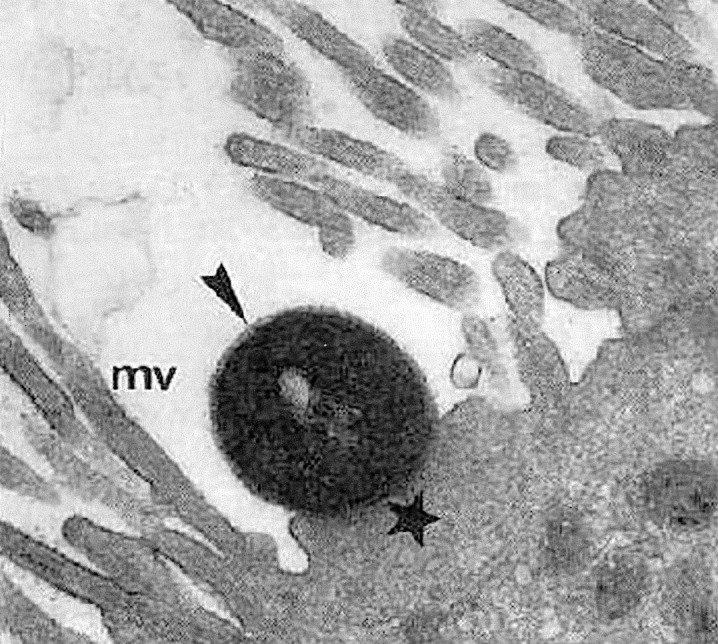
Attaching and effacing lesion showing effacement of microvilli (mv) and pedestal (star) with adherent enteropathogenic *Escherichia coli* (EPEC) (arrow). Reprinted from reference *2*, with permission of the director of American Society of Microbiology Journals.

The genetic determinants for the production of A/E lesions are located on the locus of enterocyte effacement (LEE) (*4*), a pathogenicity island that contains the genes encoding intimin, a type III secretion system, a number of secreted (Esp) proteins, and the translocated intimin receptor named Tir (*1*) (Figure 2). Two LEE insertion sites have been described on the *E. coli* chromosome, and a third unidentified insertion site has been reported (*5*).

**Figure 2:**
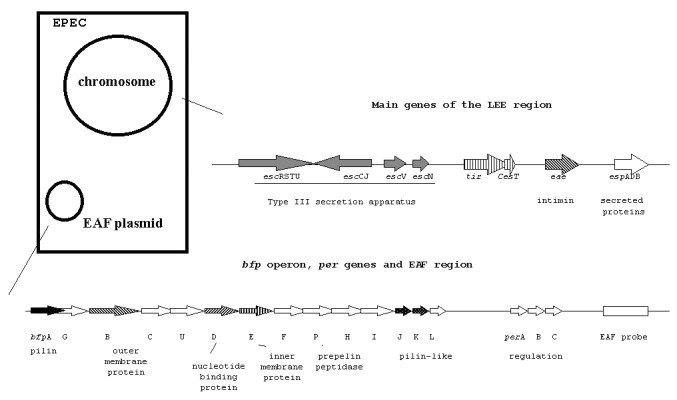
Diagram of the main genes of the locus of enterocyte effacement (LEE) region and the enteropathogenic *Escherichia coli* (EPEC) adherence factor (EAF) plasmid.

Intimin, a 94-kDa outer membrane protein encoded by the *eae* gene, is responsible for the intimate adherence between bacteria and enterocyte membranes. Studies of antigenic variations in the 280-amino acid residues of the C-terminal portion of intimin (the receptor-binding domain of the protein) and the use of polymerase chain reaction analysis allow the classification of distinct intimin types or subtypes among EPEC and STEC strains (*6*). The Esp molecules (EspA, B, and D) are involved in the formation of a translocon that delivers effector molecules to the host cell and disrupts the cytoskeleton, subverting the host cell functions (*7*). Tir, which is one of the EPEC translocated proteins, is inserted into the host cell membrane, where it acts as a receptor to intimin (*8*).

Many EPEC strains produce a characteristic adherence pattern, called localized adherence, in tissue culture cells (*9*). In this pattern, bacteria bind to localized areas of the cell surface, forming compact microcolonies (bacterial clusters) that can be visualized after bacteria have been in contact with cells for 3 hours. This phenomenon is associated with the presence of the large EPEC adherence factor (EAF) plasmid, which carries the so-called EAF sequence (Figure 2) (*1*). Also present in the EAF plasmid is the cluster of genes that encode bundle-forming pili (BFP), which interconnect bacteria within microcolonies and thus promote their stabilization (*1*).

The EAF plasmid is not essential for the formation of A/E lesions, although its presence enhances their efficiency, probably through the influence of a cluster of plasmid-borne regulatory genes (*per* *A*, *B*, *C*) that increase expression of the chromosomal LEE genes (*1*). Evidence also indicates that BFP plays a role in host cell adhesion that would similarly increase the efficiency of A/E lesion formation (*7*).

In 1995, during the Second International Symposium on EPEC in São Paulo, most participants accepted the following EPEC definition: “EPEC are diarrheogenic *Escherichia coli* that produce a characteristic histopathology known as attaching and effacing (A/E) on intestinal cells and that do not produce Shiga, Shiga-like, or verocytotoxins. Typical EPEC of human origin possess a virulence plasmid known as the EAF (EPEC adherence factor) plasmid that encodes localized adherence on cultured epithelial cells mediated by the . . . BFP, while atypical EPEC do not posses this plasmid. The majority of typical EPEC strains fall into certain well-recognized O:H serotypes” (*10*). According to this definition, the basic difference between typical and atypical EPEC is the presence of the EAF plasmid in the first group of organisms and its absence in the second.

The most studied EPEC strains belong to a series of O antigenic groups known as EPEC O serogroups. Twelve EPEC serogroups were recognized by the World Health Organization in 1987: O26, O55, O86, O111, O114, O119, O125, O126, O127, O128, O142, and O158. These serogroups include both typical and atypical EPEC strains, as well as other diarrheogenic *E. coli* categories, mainly enteroaggregative *E. coli* (EAEC) (*11*–*14*). Furthermore, most of the strains of each category correspond to specific serotypes in each O serogroup. The division of EPEC strains into typical and atypical has important implications that are not yet fully appreciated. EPEC can no longer be considered as a single group of enteropathogenic organisms. The aim of this article is to review the main differences between typical and atypical EPEC, which should be taken into account in studies involving these organisms.

## Serotypes

Typical and atypical EPEC strains belong to two different sets of serotypes (Table 1). This table was constructed on the basis of similar studies carried out in São Paulo (*11*–*15*) and the United Kingdom (*14*) and on a smaller scale in Rio de Janeiro (*16*) and Italy (*17*). Most of the typical strains were isolated in São Paulo and Rio de Janeiro and most of the atypical ones in United Kingdom and in Italy. The serotypes isolated in São Paulo include motile and nonmotile strains (indicated by placing the H antigen in brackets). The H antigens of these nonmotile strains were inferred by restriction analysis of the *fliC* genes (B.A. Botelho, et al., unpub. data). These serotypes may include both motile and nonmotile variants (Table 1).

**Table 1:** Frequently isolated enteropathogenic *Escherichia coli* (EPEC) serotypes, including typical and atypical strains

Strains	Serotypes
Typical	O55:H6, O86:H34, O111:[H2],^a^ O114:H2, O119:[H6], O127:H6, O142:H6, O142:H34
Atypical	O26:H[11], O55:[H7], O55:H34, O86:H8, O111ac:[H8], O111:H9, O111:H25, O119:H2, O125ac:H6, O128:H2

^a^ Brackets denote the frequent occurrence of nonmotile strains.

Most of the serotypes in Table 1 may easily be classified as typical or atypical. However, some serotypes are not so readily classified, mainly those that include Stx-producing strains, of which the most frequent are serotypes O26:H- and H11, and O111ac:[H8] (considered by some authors as EHEC or STEC) (*1*). In fact, these serotypes and others with properties similar to those of O128:H2 are not true atypical EPEC or STEC serotypes but rather are heterogeneous serotypes that include different clones or genetic lineages. For example, we have recently shown by random amplified polymorphic DNA that O26:H11 Stx-producing strains isolated in Europe and North America are genetically different from Stx-negative strains of the same serotype isolated in Brazil (*18*). Although this kind of study has not been done with serotype O128:H2, this serotype is also heterogeneous since it includes different ribotypes with distinct virulence characteristics (L.R. Trabulsi et al., unpub. data). Certain Stx-producing clones have an irregular geographic distribution and so may be found in some countries but not in others. Other characteristics that may complicate distinguishing typical from atypical EPEC are related to the EAF plasmid markers. For example, serotypes O119:H2 and O128:H2 react with the *bfpA* probe but do not have a true EAF plasmid. These serotypes have a 100-MDa plasmid that does not contain the *bfp* operon and consequently does not produce BFP (*19*). In contrast, some O142:H6 strains do not react with the EAF probe but produce BFP and show a typical localized adherence (LA) pattern. These strains may have an EAF plasmid with a defect in the EAF region that does not interfere with the the plasmid’s functions. Perhaps the best distinguishing characteristic for typical and atypical EPEC serotypes would be production or nonproduction of BFP.

## Virulence Characteristics

In general, typical EPEC strains are more homogeneous in their virulence characteristics than the atypical ones. With few exceptions, typical strains produce only the virulence factors encoded by the LEE region and the EAF plasmid. The exceptions are the production of the cytolethal distending toxin (CDT) by all O86:H34 strains (L.R. Trabulsi et al., unpub. data) and the production of the enteroaggregative heat stable toxin (EAST1) by some strains of serotypes O55:H6 and O127:H6 (T.A.T.Gomez et al., unpub. data) that are potential virulence factors. In contrast, atypical EPEC strains frequently express EAST1 and other potential virulence factors not encoded in the LEE region (Table 2). Accordingly, there are two kinds of atypical EPEC strains: those that express only the LEE-encoded virulence factors and those that express both LEE and the non-LEE encoded virulence factors. Usually both kinds of strains belong to a single clone (*11*,*12*,*15*). All atypical EPEC serotypes, with exception of O125ac:H6, include both kinds of strains. All strains of this serotype examined thus far show the aggregative adherence pattern and the LEE region. The occurrence of more than one kind of strain in most atypical serotypes is another interesting difference between typical and atypical EPEC.

**Table 2:** Virulence characteristics not encoded on the locus of enterocyte effacement (LEE) of atypical enteropathogenic *Escherichia coli* (EPEC) strains isolated in São Paulo, Brazil

Serotype	Characteristics
O26:[H11]^a^	EAST1, E-hly^b^
O55:[H7]	EAST1, Afa
O111ac:[H8]	E-hly
O111:[H9]	E-hly
O119:H2	EAST1
O125ac:H6	AA
O128:H2	EAST1

^a^ Brackets denote the frequent occurrence of nonmotile strains. ^b^EAST, heat-stable toxin 1 of EAEC; E-hly, EHEC hemolysin; AA, aggregative adherence; Afa, afimbrial adhesin.

**Figure 3:**
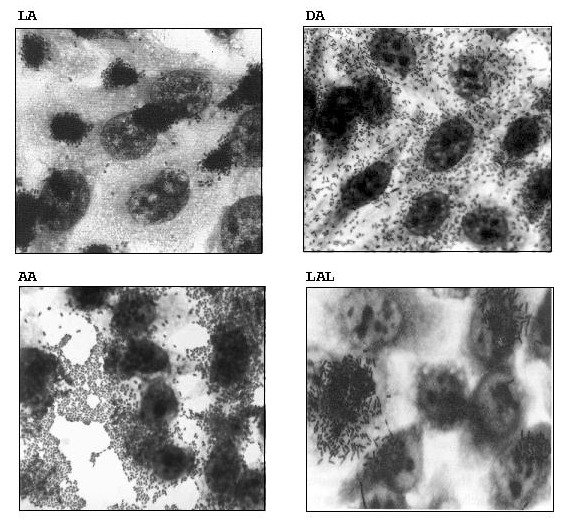
Adherence patterns of enteropathogenic *Escherichia coli* (EPEC) strains. Localized adherence (LA), diffuse adherence (DA), aggregative adherence (AA), and localized adherence-like (LAL). Magnification: X100.

Typical and atypical EPEC strains also differ in adherence patterns. The typical strains show only the LA pattern, while atypical strains may show the LAL (localized-like adherence) pattern (*12*), the DA (diffuse adherence) pattern, or the AA (aggregative adherence) pattern (Figure 3). The LAL pattern is characteristic of the strains of most serotypes and is mediated mainly by intimin (*20*). The DA pattern is mediated by the Afa adhesin (R. Keller et al., unpub. data), and the AA is mediated by an aggregative adhesin. The *cdt* gene of serotype O86:H34 (L.R. Trabulsi et al., unpub. data) and the *afa* gene of serotype O55:H7 are located on the bacterial chromosome (R. Keller et al., unpub. data). Typical and atypical EPEC also have some interesting differences with regard to the intimin types (Table 3).

**Table 3:** Intimin types of typical and atypical enteropathogenic *Escherichia coli* (EPEC) serotypes

Intimin types	Typical	Atypical
Alpha	O55:[H6],^a^ O127:H6, O142:H6, O142:H34	O111:[H9], O125ac:H6
Beta	O111:[H2], O114:H2, O119:[H6]	O26:H[11], O119:H2, O128:H2
Gamma		O55:[H7], O111ac:[H8]
Delta	O86:H34	

^a^ Brackets denote the frequent occurrence of nonmotile strains.

## Genetic Relationships

**Figure 4:**
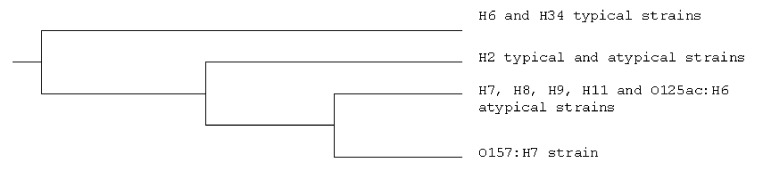
Dendogram to illustrate genetic differences between typical and atypical enteropathogenic *Escherichia coli* (EPEC) strains and *E. coli* O157:H7 strains.

To investigate the genetic relationships between typical and atypical EPEC strains, we used random amplified polymorphic DNA to study our collection of strains, which includes most of the serotypes shown in Table 1. The dendrogram derived from these data (Figure 4) shows that most typical and atypical strains belong to different genetic groups and that the atypical strains are closer to the serotype O157:H7 strains (STEC), which were included in the study for comparison purposes (S.Y.Bando et al., unpub. data). The only exceptions were the typical and atypical H2 strains that did not separate and formed a subgroup in the atypical/STEC group. The overall results of this study resemble those reported by Whittam et al. (*21*), who used multilocus enzyme electrophoresis to study a similar collection of strains and distinguished four genetic groups: EPEC 1 (H6/H34 strains), EPEC 2 (H2 strains), EHEC 1 (O55:H7 and O157:H7 strains), and EHEC 2 (O26:H11 and O111ac:H- strains). The EPEC 2 group was also closer to the EHEC groups. For this article, we have not used the division of EPEC into EPEC 1 and EPEC 2, but it may be important in the future. Several other differences exist between the two clonal groups (R. Keller et al., unpub. data). With regard to epidemiology, an EPEC 2 serotype (O111:H2) is strongly associated with nosocomial infection, while an EPEC 1 serotype (O119:H6) is more strongly associated with infection in the community (*22*).

## Pathogenicity

The pathogenicity of most typical EPEC serotypes has been confirmed by volunteer studies (*1*). For atypical EPEC we are aware of only one volunteer study, which was performed by Levine et al. (*23*) with an O128:H2 strain. This strain was administered in differing doses to 15 adult volunteers, none of whom became ill. Although this study was carefully conducted, its results are difficult to evaluate because the virulence characteristics of the strain were not known and serotype O128:H2 may include nonvirulent strains (*24*).

 The atypical EPEC strains may be less virulent than the typical ones. One reason may be the lack of the EAF plasmid; Levine et al. (*25*) have shown that an O127:H6 strain without plasmid was less virulent for adult volunteers than the wild-type strain. However, atypical EPEC strains have not been proven to be less pathogenic, and these organisms have other virulence factors that may compensate for the absence of the EAF plasmid. More studies are necessary to resolve this issue.

## Association with Diarrhea

Typical EPEC serotypes are strongly associated with diarrhea in children <1 year of age. In this age group, these serotypes have been found to be the main cause of endemic diarrhea in several well-controlled studies carried out in Brazil (*26*,*27*). The frequency of typical EPEC serotypes in children >1 year of age is lower and similar to the frequency in controls (2%-4%). Adult infections are rare and usually associated with other conditions (*1*). The increased resistance in older children and adults may be associated with the development of immunity or the loss of receptors for some specific adhesin (*1*).

Regarding immunity, several studies carried out in Brazil (*28*) and more recently in Mexico (*29*) have shown that children develop high levels of antibody against the main EPEC virulence factors. In addition, the colostrum of mothers living in endemic areas is very rich in immunoglobulin A antibodies against the EPEC virulence factors (*28*–*30*). Much less is known about the association of atypical serotypes with diarrhea, but usually these serotypes are isolated from children with diarrhea who are not carriers of other enteropathogenic agents. A strong association of atypical EPEC serotypes with endemic diarrhea has not yet been demonstrated. However, a large outbreak of diarrhea caused by serotype O111:H9 has been described in Finland (*31*).

## Prevalence in Developing and Industrialized Countries

A remarkable epidemiologic difference between typical and atypical EPEC serotypes is their geographic distribution. Typical EPEC serotypes have traditionally been associated with outbreaks of infantile diarrhea, and, in fact, the first EPEC strains isolated in different countries were of serotypes O55:H6 and O111:H2 (*32*). In the past, these epidemic serotypes were frequently identified in industrialized countries as a cause of outbreaks and sporadic cases of diarrhea, but at present they are very rare (*1*). In these countries today, serotypes without the EAF plasmid predominate (*14*,*33*). In the United Kingdom, for example, EAF-positive strains represent only 10% of all EPEC strains (*14*). The situation in developing countries is not well defined, but several studies in Brazil in the 1980s and early 1990s showed a high frequency of typical serotypes (*34*,*35*). However, some recent studies have shown a very low frequency of typical EPEC and a relatively high frequency of atypical EPEC (L.C. Campos, pers. commun. and unpub. data). This finding coincides with a decline in the number of diarrheal cases in several regions in Brazil, suggesting that the changes that have occurred in industrialized countries are likely already under way in Brazil. The reason for these changes is not clear, but the decline in the frequency of the EAF-positive serotypes that has occurred in Europe and the United States and is beginning to occur in Brazil may be due to improvements in therapy, sanitary conditions, and control of hospital infections. On the other hand, the emergence and rise in frequency of atypical EPEC strains may have origins similar to those that led to the emergence and increase in frequency of O157:H7 and other STEC serotypes (*35*).

## Reservoir

Typical EPEC serotypes have not been found in animals (*1*), suggesting that humans are the only living reservoir for these organisms. In contrast, most atypical EPEC serotypes have been isolated from different animal species. The association between serotype O26:H11 and calves is well known (*36*). Recent studies have emphasized the isolation of Stx-producing strains because of their role in hemolytic uremic syndrome, but *eae*-positive, Stx-negative strains have been isolated from cattle (*37*). This kind of strain should be considered atypical EPEC. A similar situation exists in regard to serotype O111ac, and the 69 O111ac strains reported by Ewing et al. in 1963 were all isolated from monkeys (*38*).

Serotype O128:H2 is rather frequent in rabbits and dogs and, like the human strains isolated in Brazil, is EAF negative (Pestana de Castro, pers. commun.). In a recent study by Pestana de Castro’s group, serotypes O119:H2 and O111:H25 (an EAF-negative serotype rare in Brazil but frequent in the United Kingdom) were isolated frequently from dogs. More studies of the prevalence of atypical EPEC serotypes in animals are needed, but available data strongly suggest that the primary reservoir for these organisms is different animal species, as is the case with STEC strains.

## Stx-Negative and *eae*-Positive *E. coli* Strains in Non-EPEC O Serogroups

Both stx-negative and *eae*-positive *E. coli* strains are found in many non-EPEC O serogroups (*39*). We have detected such strains in more than 30 *E. coli* O serogroups, and a large proportion of strains do not agglutinate in the usual set of *E. coli* O antisera. Some strains react with the EAF probe (*eae*+, EAF+ strains), but most do not react with this probe (*eae*+, EAF- strains). With a few exceptions, only one or two strains of each of these serotypes have been reported (*40*).

The additional virulence characteristics of the *eae*+, EAF+ strains have not been studied, but recently we investigated the virulence profile of 49 different *eae*+, EAF- strains isolated from children with diarrhea in São Paulo. The profile was similar to that of atypical EPEC: many strains were EAST1+ and *E-hly*+, and a few expressed either the AA or the DA adherence pattern. Some strains had the gamma intimin sequence, and in many of the strains the intimin type could not be identified.

Some of these strains do correspond to typical or atypical EPEC, and more studies are necessary to establish a precise concept for them, especially for the EAF-negative strains. Some are likely STEC strains that have lost the *stx* genes; we cannot exclude the possibility that the DA and AA strains are not true EAEC or DAEC that have received the LEE pathogenicity island by horizontal transfer. The situation is quite different for atypical EPEC, since a larger number of strains have been studied and most of them belong to well-characterized serotypes.

The role played by these EAF+ and EAF- strains outside the EPEC serogroups in endemic diarrhea has not been established. In general, the strains are rarely isolated from diarrheal cases and controls, and the global difference is not statically significant. However, some *eae*+, EAF+ serotypes as well as some *eae*+, EAF- strains with specific virulence profiles seem to be associated with endemic diarrhea (*2*,*33*,*40*). With regard to outbreaks, an *eae*+, EAF- serotype (O39:H-) was responsible for a foodborne diarrheal outbreak in 1991, involving 100 adults in Minnesota (*41*).

## Conclusion

Typical and atypical EPEC seem to constitute two groups of distinct organisms that have in common the LEE pathogenicity island. Atypical EPEC are closer to STEC in genetic characteristics, serotypes, production of toxins, reservoir, and other epidemiologic aspects. As STEC, they resemble emerging pathogens. In industrialized countries, they have become a more frequent cause of diarrhea than typical EPEC, and the same shift may be occurring in Brazil. A large number of Stx-negative, *eae*-positive typical and atypical EPEC-like strains outside the EPEC O serogroups, as well as atypical EPEC strains, require further study in regard to their virulence and epidemiologic significance.
